# A Novel Application of Spinning Disk Technology to Collect Plasma from Whole Blood Prior to Quantifying Plasma RNA

**DOI:** 10.17912/micropub.biology.001007

**Published:** 2023-11-03

**Authors:** Naomi Rapier-Sharman, Mae-Lynn L. Hutchinson, Carlos M. Moreno, Abraham Quaye, Brian D. Poole, K. Scott Weber, William G. Pitt, Brett E. Pickett

**Affiliations:** 1 Department of Microbiology and Molecular Biology, Brigham Young University, Provo, Utah, United States; 2 Department of Chemical Engineering, Brigham Young University, Provo, Utah, United States

## Abstract

The spinning disk technology has previously been utilized to isolate bacterial components from blood in hours instead of days. We hypothesized that this platform could be applied as an alternative approach to isolating plasma RNA from a whole blood sample. We consequently tested the efficacy of the spinning disk technology to extract plasma from whole blood upstream of RNA isolation and analysis. To do so, we collected plasma using either the spinning disk or the typical two-spin centrifuge method. We found that the spinning disk method results in significantly more hemolysis during collection than the conventional two-spin centrifuge method. However, when plasma RNA recovered from both collection methods was quantified using quantitative Real-Time Polymerase Chain Reaction (qRT-PCR), we found that the spinning disk method yielded a higher plasma RNA concentration than the two-spin centrifuge method. This suggests that the spinning disk may be an efficient alternative method to recover plasma RNA. Further work is needed to determine whether red blood cell RNA contamination is present in the plasma RNA extracted from spinning disk-processed plasma.

**Figure 1.**
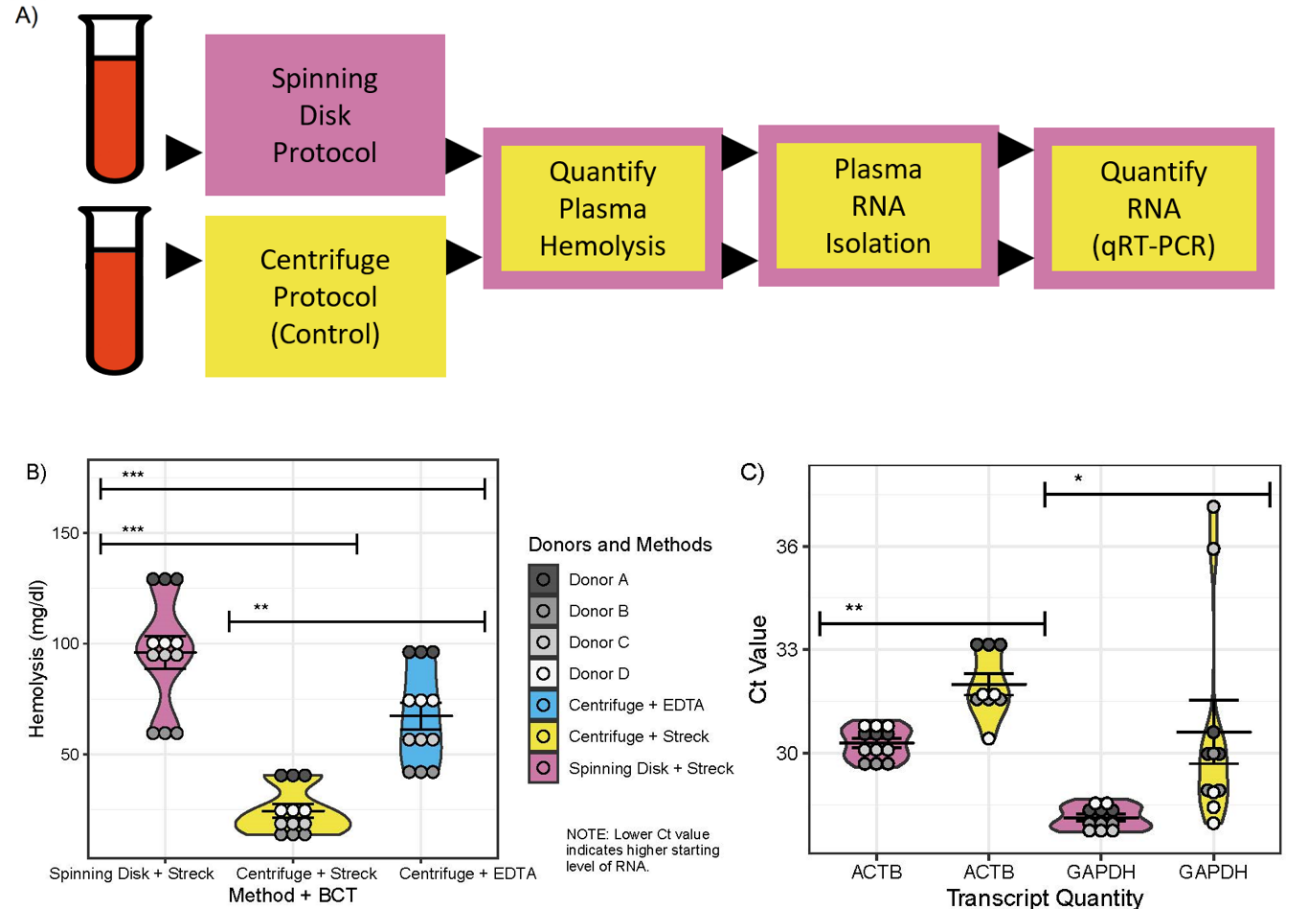
**1A. Analytical Workflow Evaluating the Spinning Disk Method for Plasma RNA Isolation.** A visual representation of the comparative workflow used to test the spinning-disk method (test, pink boxes) and two-spin centrifuge method (control, yellow boxes) using blood collected in Streck Complete RNA (Streck) tubes. Samples drawn in EDTA tubes (not included in the workflow figure), were treated with the two-spin centrifuge method and evaluated for hemolysis, but not evaluated for RNA quantity. **1B. Hemolysis Profiles of Plasma Samples Isolated with Different Methods.** Hemolysis was measured in plasma extracted from blood, which were collected in either Streck tubes or EDTA tubes, and treated with either the spinning disk method or the two-spin centrifuge method. Three experimental groups, which combined different plasma extraction methods and blood collection tubes (BCT), were evaluated including: Spinning Disk + Streck (pink), Centrifuge + Streck (yellow), and Centrifuge + EDTA (blue). Gray-scale dots are shaded by donor (biological replicate) and represent three technical replicate measurements per donor (biological replicate). Significant differences in hemolysis between groups (t-test) are indicated. ** = p-value < 0.01. *** = p-value < 0.001. **1C. Plasma RNA Quantity in Spinning Disk- or Centrifuge-Processed Streck Samples.** Average plasma RNA abundance quantified using qRT-PCR cycle threshold (Ct) values for the experimental groups (Spinning Disk + Streck, Centrifuge + Streck). Violin plots are colored by experimental group, with Spinning Disk + Streck in pink and Centrifuge + Streck in yellow. Gray-scale dots are shaded by donor (biological replicate) and represent three technical replicate measurements per donor (biological replicate). Significant differences in the distribution between groups are indicated (t-test). * = p-value < 0.05. ** = p-value < 0.01.

## Description

The spinning disk method has primarily been used to separate blood components prior to bacterial DNA extraction and high-speed antibiotic resistance profiling (~2-4 hours instead of 3-5 days using traditional bacterial culture methods) (Hoj et al., 2021; Meena et al., 2020) . Briefly, blood is collected into a 10 mL tube, followed by 3-6 mL of whole blood being diluted to reach a final volume of 8.5 mL at a hematocrit of 32. The spinning disk utilizes centrifugal force to trap red and white blood cells from the diluted blood sample in outer wells, while plasma is allowed to escape into interior wells. The separated plasma can then be pipetted out to obtain ~4 mL of plasma per spin. Current analytical workflows for extracting plasma RNA generally include the following steps: blood collection into specialized tubes, immediate separation of blood components via centrifuge, plasma RNA isolation using guanidium- or silica column-based RNA extraction kits, and RNA quantification by various methods (Tzimagiorgis et al., 2011) . Plasma RNA is currently used to help detect and monitor pregnancy complications such as pre-eclampsia (Pan et al., 2017; Rasmussen et al., 2022) . Additionally, plasma RNA is a useful tool for diagnosing and monitoring viral infections (Pan et al., 2017) . The purpose of this study was to determine efficacy of the spinning disk as an upstream blood component separation step before plasma RNA extraction and quantification, and to compare the results to the typical upstream two-spin centrifugation method.

**Blood Collection and Component Separation:** To compare these two plasma separation methods, we collected blood from four donors (biological replicates), in sets of 3 tubes per donor. Each donor set included two Streck RNA Complete BCT (Streck) Tubes and one K3-EDTA (EDTA) tube ( Figure 1A ). We paired samples from each donor set when performing downstream statistical analyses. From each donor set, plasma was collected using the spinning disk method (test) from one Streck tube and using the traditional two-spin centrifugation method (control) from the other Streck tube. To enable downstream comparisons between the methods and blood collection tubes (BCTs), we additionally collected and analyzed plasma using the traditional two-spin centrifugation step from commonly-used EDTA tubes.

**Plasma Hemolysis Evaluation:** To determine the level of background hemolysis in the collected plasma, we used a spectrophotometric-based method, the Harboe Method with the Allen Correction. This method quantifies free oxyhemoglobin levels, a marker of hemolysis which rises in proportion to total hemolysis (Malinauskas, 1997) . We subsequently compared the hemolysis present in plasma samples from different methods, namely, Spinning Disk + Streck, Centrifuge + Streck, and Centrifuge + EDTA, using paired t-tests on sample measurements from four donors (biological replicates) with three technical replicates per donor ( Figure 1B ). We found that the Spinning Disk + Streck group had significantly more hemolysis than the Centrifuge + Streck group (p < 0.001), that the Spinning Disk + Streck group had significantly more hemolysis than the Centrifuge + EDTA group (p = 0.007), and that the Centrifuge + EDTA group had significantly more hemolysis than the Centrifuge + Streck group (p < 0.001). EDTA tubes were not included in further analyses due to poor preservation of mRNA by K3-EDTA tubes and EDTA’s known PCR-inhibitory properties (Rainen et al., 2002; Schrader et al., 2012) .

**Plasma RNA Isolation & Quantification:** To evaluate the efficacy of the spinning disk plasma separation method, we next determined whether the plasma RNA yield differed between the Streck + Spinning Disk and Streck + Centrifuge groups. To do so, we isolated plasma RNA from all Streck tube samples using the QIAamp Circulating Nucleic Acids Kit (Qiagen), after which we quantified the plasma RNA extracted from each sample using quantitative Real-Time Polymerase Chain Reaction (qRT-PCR). To do so, for samples from four donors (biological replicates) with three technical replicates per sample, we measured the ACTB and GAPDH housekeeping genes according to a standard quantification procedure for low-concentration RNA such as plasma RNA (Tzimagiorgis et al., 2011) . We then compared the cycle threshold (Ct) values from each extraction workflow, where higher Ct values indicate a lower initial concentration of RNA, using a paired t-test. This analysis revealed that the Spinning Disk + Streck group had a significantly higher plasma RNA yield than the Centrifuge + Streck group for both plasma GAPDH transcripts (p = 0.022) and plasma ACTB transcripts (p = 0.005), as shown by the lower Ct values in the Spinning Disk + Streck samples ( Figure 1C ). We also observed that variability in pipetting technique does not contribute significantly to sample hemolysis. We anticipate that additional work will be required to reduce spinning disk hemolysis, potentially including reducing red blood cell impact against the exterior disk wall by utilizing a slower ramp-up acceleration. We expect that this modification could reduce hemolysis in spinning disk plasma. We see at least two possible reasons for the significant difference between methods in recovered plasma RNA quantity. The first possibility is that the spinning disk method could be more effective at plasma RNA recovery than the two-spin centrifuge method. The second possibility is that the observed rise in spinning disk plasma RNA concentration could be due to higher contamination by cellular RNA released by hemolysis. These results should be considered as preliminary until tests are done to evaluate the presence of hemolytic/RBC-specific RNA biomarkers within the extracted plasma RNA. Potential methods for evaluating RBC RNA presence in plasma RNA include measuring the miR-23a/miR-451 proportion to quantify RBC lysis (Blondal et al., 2013) . miR-16 and miR-144 have also been reported as reliably dynamic plasma RNA markers of hemolysis (Kolenda et al., 2020) .

## Methods

**Sample Collection and Experiment Design:** IRB approval was obtained before collecting whole blood samples (Brigham Young University, Protocol # X2021-135). Blood was drawn into a set of two Streck RNA Complete BCT (Streck) Tubes and one K3-EDTA (EDTA) tube per donor (Becton Dickinson Hemogard™ #366643). Hematocrit was measured from two technical replicates per donor and blood from each donor was diluted with phosphate-buffered solution (PBS) to a standard hematocrit of 32 to ensure uniform performance of the spinning disk. One of the two Streck tubes collected from each donor was processed using the spinning disk method, while the second of the two Streck tubes from each donor was processed using the two-spin centrifuge method. The EDTA tube from each donor was also processed using the two-spin centrifuge method to provide an additional datapoint on tolerated hemolysis levels in typical hospital/clinical lab samples.

The centrifugation treatment consisted of a two-spin protocol, with a 15-minute primary spin at 1800 relative centrifugal force (RCF) followed by a 15-minute clarifying spin at 2800 RCF, both in the high-speed centrifuge according to Streck manufacturer protocol as previously reported, after which the plasma layer was carefully collected for further analysis (N. M. George et al., 2020). The spinning disk method we used consisted of a primary spin on the spinning disk and a subsequent clarifying microcentrifuge spin. Before beginning the procedure, the spinning disk and its lid were both sterilized by using a modified version of the protocol used in past work (Buchanan et al., 2017) . Briefly, the disk was soaked in 10% bleach for one minute, after which it was dried using compressed air. The lid was then sterilized by spraying with 70% ethanol and drying with compressed air. After depositing 8.5 mL of PBS-adjusted blood (to a hematocrit of 32) into the center of the spinning disk, the disk was accelerated from 0 revolutions per minute (RPM) to 3,000 RPM in 5 seconds and was held at 3,000 RPM (604 RCF) for 30 seconds. The disk was then carefully decelerated until it reached 0 RPM (Wood et al., 2019) , after which the plasma layer was carefully pipetted from the wells of the spinning disk into microcentrifuge tubes for a 10-minute clarifying microcentrifuge spin at 3700 RCF. Although the clarifying microcentrifuge spin was not part of the spinning disk protocol for isolating bacteria, this step was added to remove any remaining red blood cells, white blood cells, or platelets from the plasma before measuring hemolysis.

**Plasma Hemolysis Evaluation:** The plasma was carefully collected for further analysis from the Streck tubes using either the spinning disk method or two-spin centrifugation method, and from the EDTA tubes using the two-spin centrifugation method. Plasma hemolysis was then determined via spectrophotometry by measuring the absorption at wavelengths of 415, 380, and 450 nm in triplicate and applying the Harboe method with the Allen correction according to the following equation: Hb = (1 mg/dL)(167.2(A415) - 83.6(A380) - 83.6(A450)) wherein A415 indicates absorbance at 415 nm, etc. (Malinauskas, 1997) . A comparison of the hemolysis levels in plasma separated with one of: Spinning disk + Streck, two-spin centrifugation + Streck, and two-spin centrifugation + EDTA were performed using a paired t-test in Microsoft Excel and plotted using ggplot2 (Wickham, 2011) . EDTA tube samples were not included in further testing due to poor preservation of mRNA by K3-EDTA tubes and EDTA’s known PCR-inhibitory properties (Rainen et al., 2002; Schrader et al., 2012) .

**Plasma RNA Extraction and Quantification:** Following plasma separation using either the spinning disk or the centrifuge method, plasma RNA was extracted from all Streck tube samples according to the manufacturer’s instructions found in the Cell-free RNA next-generation sequencing workflow for the Streck RNA Complete BCT document (N. George, 2018; N. M. George et al., 2020). In brief, plasma RNA was extracted from 3 mL of sample using the QIAamp Circulating Nucleic Acids Kit (Qiagen) according to the microRNA protocol for 3 mL samples with following adjustments: 1) extending the incubation step from 30 minutes to 60 minutes at 59°C, and 2) eluting with 87.5 μL of buffer AVE. DNase I digestion was then immediately performed with the RNase-Free DNase Set (Qiagen) according to the Appendix B protocol in the QIAamp Circulating Nucleic Acids handbook, with the adjustment of eluting with 18 μL of RNAse-free water. RNA cleanup was then performed using the RNeasy MinElute Cleanup Kit according to the QIAamp Circulating Nucleic Acids Handbook Appendix C protocol. Following cleanup, the concentration of each plasma RNA sample was checked in triplicate using a Thermo Nanodrop instrument to obtain the 280/260 and 230/260 absorbance ratios, to measure sample purity and estimate RNA concentration. After determining RNA concentrations, purified RNA samples were stored in a -80°C freezer. The RNA samples were prepared for qRT-PCR using the High Capacity RNA-to-cDNA Kit (Applied BioSystems) with random hexamer primers to create first-strand synthesis cDNA libraries prior to storage at -80°C. qRT-PCR was performed on these cDNA libraries on a StepOnePlus Real-Time PCR System (Applied Biosystems) using the SYBR GreenER qPCR SuperMix Universal kit (Invitrogen) and the following primers: GAPDH forward primer 5’- AGCCGAGCCACATCGCT-3’, GAPDH reverse primer 5’-TGGCAACAATATCCACTTTACCAGAGT-3’ (Lossos et al., 2003) , ACTB forward primer 5’-CCCCGCGAGCACAGA-3’, ACTB reverse primer 5’-CCACGATGGAGGGGAAGAC-3’ (Lossos et al., 2003) . Following the qRT-PCR run, results were downloaded from the StepOnePlus software for statistical analysis. Ct values for GAPDH and ACTB were generated for samples prepared with the spinning disk or two-spin centrifugation-only methods. The statistical significance for the values for each procedure were then generated using a paired t-test in Microsoft Excel and plotted using ggplot2 (Wickham, 2011) .

## Extended Data

Description: The complete dataset of measurements found during this study and analyzed in this paper.. Resource Type: Dataset. DOI: 10.22002/mcr98-ays65
